# Development of Biomimetic Hepatic Lobule-Like Constructs on Silk-Collagen Composite Scaffolds for Liver Tissue Engineering

**DOI:** 10.3389/fbioe.2022.940634

**Published:** 2022-06-23

**Authors:** Lina Guo, Ziqing Zhu, Chuanzhou Gao, Kaiwen Chen, Shenzhou Lu, Hexin Yan, Wenming Liu, Mingqi Wang, Yanfang Ding, Lin Huang, Xiuli Wang

**Affiliations:** ^1^ College of Basic Medical Science, Dalian Medical University, Dalian, China; ^2^ National Engineering Laboratory for Modern Silk, College of Textile and Clothing Engineering, Soochow University, Suzhou, China; ^3^ School of Bioengineering, State Key Laboratory of Fine Chemistry, Dalian University of Technology, Dalian, China; ^4^ Department of Anesthesiology and Critical Care Medicine, Renji Hospital, Shanghai Jiaotong University School of Medicine, Shanghai, China; ^5^ Shanghai Engineering Research Center of Peri-operative Organ Support and Function Preservation, Shanghai, China; ^6^ General Surgery Center, Department of Hepatobiliary Surgery II, Zhujiang Hospital, Southern Medical University, Guangzhou, China

**Keywords:** hepatic lobule, silk fibroin, collagen, liver tissue engineering, dynamic culture, biomimetic, transplantation

## Abstract

Constructing an engineered hepatic lobule-mimetic model is challenging owing to complicated lobular architecture and crucial hepatic functionality. Our previous study has demonstrated the feasibility of using silk fibroin (SF) scaffolds as functional templates for engineering hepatic lobule-like constructs. But the unsatisfactory chemical and physical performances of the SF-only scaffold and the inherent defect in the functional activity of the carcinoma-derived seeding cells remain to be addressed to satisfy the downstream application demand. In this study, SF-collagen I (SFC) composite scaffolds with improved physical and chemical properties were fabricated, and their utilization for bioengineering a more hepatic lobule-like construct was explored using the immortalized human hepatocyte-derived liver progenitor-like cells (iHepLPCs) and endothelial cells incorporated in the dynamic culture system. The SFC scaffolds prepared through the directional lyophilization process showed radially aligned porous structures with increased swelling ratio and porosity, ameliorative mechanical stiffness that resembled the normal liver matrix more closely, and improved biocompatibility. The iHepLPCs displayed a hepatic plate-like distribution and differentiated into matured hepatocytes with improved hepatic function *in vitro* and *in vivo*. Moreover, hepatocyte–endothelial cell interphase arrangement was generated in the co-culture compartment with improved polarity, bile capillary formation, and enhanced liver functions compared with the monocultures. Thus, a more biomimetic hepatic lobule-like model was established and could provide a valuable and robust platform for various applications, including bioartificial liver and drug screening.

## Introduction

Constructing three-dimensional (3D) liver models with biomimetic structure and function is of great interest owing to its promisingly potential applications in clinical treatment, fundamental research, and drug screening. Unfortunately, desirable hepatocyte cultivation *in vitro* is severely hindered by deficiency technology to fully recapitulate the native growth conditions. Co-culture with multiple nonparenchymal cells and/or incorporation with different extracellular matrixes (ECM) has been widely explored to improve the viability, maturation, and function of hepatocytes (Cuvellier et al., 2021; Das et al., 2020; Liu et al., 2021). Nonetheless, few investigations have focused on precisely controlling hepatic tissue architecture at the microscale, although both the complex architecture and specific organization of hepatic lobules play an important role in determining the phenotype and function of the actual liver tissue. It is known that native human hepatocytes organize into hepatic irregular plates that radiate toward a central vein to form polygonal hepatic lobules. This unique architecture provides a foundation for the high integration of hepatocytes and their surrounding microenvironment, which promotes the generation of bile capillaries and enhances its adequate spatial and temporal contact with blood for mass transport.

Motivated by the goal of recreating more histologically and physiologically relevant hepatocyte culture systems *in vitro*, different strategies, such as decellularized liver scaffolds (Uygun et al., 2010; Watanabe et al., 2019), micro-fluidic chips (Ho et al., 2013; Ya et al., 2021), and 3D printing methods (Kang et al., 2020), have been employed to reconstruct a liver biomimetic architecture, and the biomimetic configuration has been confirmed to positively affect hepatocyte morphology, polarity, viability, and maturation. However, most methods are still limited by uncontrollable cell localization, pseudo-3D cultures, or complex construction processes that might lead to cell damage. Recently, our laboratory reported the fabrication of lobule-like silk fibroin (SF) scaffolds using a radially directional freezing strategy (Wang et al., 2022). By co-culturing hepatocytes and endothelial cells on this SF scaffold, a more biomimetic human hepatic lobule-like culture model with improved phenotype and function was developed *in vitro*. This study strongly supports the important role of matrix architecture in determining cell behavior and hepatic tissue reconstruction.

Although our previous study has proven encouraging for the construction of a lobule-mimetic culture model *in vitro*, we are nonetheless faced with certain limitations. On the one hand, due to the lack of integrin-mediated cell-binding sites, cell adhesion to SF scaffolds was less desirable, mainly relying on low-affinity cell-binding domains (carboxy-terminal arginine residues or electrostatic interactions between cells and scaffolds) (Maghdouri-White et al., 2014; Ruoslahti and Pierschbacher, 1987). In addition, SF-only scaffolds possess relatively high mechanical properties. For example, our previous study demonstrated that the modulus of the scaffold prepared with a 6% (w/v) SF solution was 67.3 ± 2.0 kPa, which was significantly higher than that of the normal liver (approximately 2.8 ± 7.4 kPa). On the other hand, the immortalized hepatocarcinoma C3A cells used before were inadequate in replicating the native hepatic phenotype and function due to their insurmountable inherent defects associated with cancerous phenotype and impaired functionality. Their immortalized activity poses a risk of carcinogenesis once implanted *in vivo*, hindering not only the model-based exploration of the complex interactions between hepatocytes and the microenvironment but also the downstream application for *in vivo* implantation.

Studies have demonstrated that mixing SF solutions with natural biomolecular polymers could overcome some drawbacks of SF scaffolds (Buitrago et al., 2018; Goczkowski et al., 2019). Type I collagen, a predominant subtype synthesized in connective tissue, is the most abundant collagen in human liver tissues with rich integrin-mediated cell-binding sites (Martinez-Hernandez and Amenta, 1993; Willemse et al., 2020; Kumaran et al., 2005). Importantly, several precedents for using collagen as a biomaterial either in the hepatocyte culture *in vitro* or the clinical settings have been noted (Rico-Llanos et al., 2021; Serna-Márquez et al., 2020), in which collagen provides biomimetic matrix support for the 3D culture of mammalian cells. Hence, given these apparent advantages of collagen over the existing culture system, its incorporation in the 3D culture compartment may benefit to establish a lobule-mimetic culture model with improved phenotype and function.

Therefore, in the present study, an SF-collagen (SFC) composite scaffold was fabricated by directional lyophilization with an SF-collagen type I mixed solution. Meanwhile, more functional primary human hepatocyte-derived liver progenitor-like cells (iHepLPCs) were employed to replace the immortalized hepatocarcinoma C3A cells before. We hypothesized that the primary iHepLPCs growing in a 3D microenvironment provided by the stromal cells, ECM molecules, and SFC composite scaffold with unique lobule-like architecture not only generate liver tissue-like structures that more closely resemble the *in vivo* hepatic lobule morphology but also contribute to producing an optimized 3D biomimetic lobule-like culture model with improved differentiated functionality. Toward this goal, the reconstructed 3D culture model was characterized by its growth profile, histology, and gene expression. Moreover, its potential capability for transplantation was evaluated through subcutaneous implantation in nude mice.

## Experimental Methods

### Cell Maintenance Culture and Differentiation

The human hepatoma cell line C3A (American Type Culture Collection, Manassas, VA, United States) was cultured in Eagle’s minimum essential medium (Gibco, Carlsbad, CA, United States). The complete medium contained 10% fetal bovine serum (FBS, ScienCell Research Laboratory, Carlsbad, CA, United States) and 1% penicillin/streptomycin solution (P/S, Solarbio, Peking, China). Professor Hexin Yan of the Shanghai Cancer Institute (Shanghai, China) provided the iHepLPCs, GFP-transfected iHepLPCs, transition and expansion medium (T&EM), and hepatic maturation medium (HMM). The detailed transfection method and components of both mediums can be found in a previous study (Wang et al., 2019). The cells were maintained and expanded in T&EM and passaged at a ratio of 1:3 after dissociation in TrypLE solution (Gibco). Subsequently, the iHepLPCs were maintained in T&EM until they attained 90% confluence for rapid hepatic differentiation. Then, the medium was replaced with HMM, and the cells were cultivated for an additional 7–10 days to allow for complete differentiation into iHepLPC-derived hepatocytes (iHepLPC-Heps). Alternatively, human umbilical vein endothelial cells (HUVECs, ScienCell) were cultured in an endothelial growth medium (EGM, ScienCell) and used between passages three and six. All the cells were incubated in a humidified incubator containing 5% CO_2_ at 37°C, and the medium was changed every other day.

For iHepLPCs and HUVECs co-culture experiments, a combined medium composed of T&EM/HMM and EGM at a volume ratio of 2:1 was used, which had previously been tested to ensure the proper growth of each cell type under monolayer culture conditions.

### Preparation of SF Solution

Soochow University (Suzhou, China) kindly provided SF aqueous stock solution. Briefly, SF fibers isolated from the cocoons of a *Bombyx mori* silkworm were boiled for 30 min in an aqueous solution of 0.02 M Na_2_CO_3_ (Solarbio). Subsequently, the fibers were rinsed thoroughly with distilled water to extract the glue-like sericin proteins. After air-drying, the degummed silk fibers were placed in a 9.3 M lithium bromide (Aladdin Bio-Chem Technology, Shanghai, China) solution in a glass beaker at 60°C for 4–5 h. Then, the solution was dialyzed against distilled water in a dialysis bag with a molecular weight cut-off of 3500 Da for 3 days. Finally, the solution was centrifuged, and the concentration was determined by weighing the residual solid of a known solution volume after drying at 60°C.

### Fabrication of SFC Composite Hepatic Lobule-like Scaffolds

The composite biomimetic hepatic lobule-like silk scaffolds were fabricated following our previous study with minor modifications (Supplementary Figure S1) (Wang et al., 2022). To prepare the SFC composite scaffolds, 6% SF solution (w/v) was mixed with 2 mg/ml rat tail collagen I solution at a ratio of 5:1 and 2:1 to keep the SF concentration at 5 and 4%; 5 and 4% SF solution served as the control (Supplementary Table S1). After complete blending, SFC and SF solutions at different concentrations were transferred into 15-ml centrifuge tubes and rapidly immersed in liquid nitrogen for 30 min in an upright position. Next, the frozen solutions were lyophilized for 48 h and then autoclaved to induce SF β-sheet structure formation. Finally, ready-to-use scaffolds with a thickness of 1–2 mm, an outer diameter of 6 mm, and an inner diameter of 1 mm were prepared.

### Performance Testing of SFC Composite Hepatic Lobule-like Scaffolds

#### Swelling Ratio

The scaffolds were immersed in ultrapure water at room temperature for 24 h. After excess water was removed, the wet weight of the scaffolds (Ws) was determined. The samples were then dried overnight in an oven at 60°C under vacuum, after which the dry weight of the scaffolds (W_d_) was determined. By running the samples of each group in triplicate, the swelling ratio of the scaffolds was calculated as follows:

$$\text{Swelling ratio}=\left(\left({\text{W}}_{\text{s}}-{\text{W}}_{\text{d}}\right)/{\text{W}}_{\text{d}}\right)\times 100\%.$$

#### Porosity

A scaffold of weight W_1_ (in g) and volume V (in cm^3^) was immersed in hexane (Sigma-Aldrich, St. Louis, MO, United States) for 10 min at room temperature. After removing excess liquid from the surface, the total weight of the scaffold was recorded as W_2_ (g). The density of hexane is 0.66 g/cm^3^. Subsequently, by running each sample in triplicate, the porosity of the scaffolds was determined using the following formula:

$$\text{Scaffold porosity}=\left({\text{W}}_{2}-W_{1}\right)/0.66\text{V}\times 100\%.$$

#### *In Vitro* Degradation

Dried cylindrical scaffolds (8 mm in diameter and 2 mm in height) with a weight of W_0_ (g) were incubated and immersed in 1 ml of PBS solution with or without 6 U/ml protease XIV (Sigma-Aldrich) for 2, 4, 6, and 8 days. The samples were incubated at 37°C, after which the medium was changed every 2 days. At each time point, the scaffolds were dried at 60°C and weighed (W_n_) (g). By running the samples in triplicate, the remaining mass fraction of the scaffolds *in vitro* was calculated using the following formula:

$$\text{Remaining mass fraction}=\left({\text{W}}_{\text{n}}/{\text{W}}_{0}\right)\times 100\%.$$

#### Mechanical Properties

We determined mechanical strength using cylindrical scaffolds (8 mm in diameter and 8 mm in height) immersed in ultrapure water for 12 h at room temperature. The compression properties of the scaffolds were evaluated at room temperature using an Instron 3365 testing frame (Instron, Norwood, MA, United States) with a 100 N loading cell. The load was applied until the scaffold had compressed to 80% of its original height. Subsequently, the pressure was released. The compressive modulus was calculated from the linear elastic region in the stress–strain curve, and all data were analyzed as averages of four to six tests, comprising three parallel scaffolds.

#### Fourier Transform Infrared Spectroscopy (FTIR)

The secondary structures of the scaffolds were analyzed using a Nicolet FTIR 5700 spectrometer in the attenuated total reflection mode (Thermo Scientific, FL, United States). Briefly, a thin layer of the SF scaffold with a thickness of 1–2 mm was cut and subjected to FTIR analysis. Sixty-four scans were recorded for each measurement, with a resolution of 4 cm^−1^ and wavenumber of 400–4000 cm^−1^. The samples were run in triplicate in each group.

### 3D Culture on Silk Scaffolds

Before cell inoculation, the ready-to-use scaffolds were autoclaved for sterilization and then pre-coated with a growth factor reduced-Matrigel^™^ (0.15 mg/ml; BD Biosciences, SanJose, CA, United States) diluted using a culture medium to facilitate cell attachment. Then, to construct the human-engineered liver lobule-like cultures, iHepLPCs and HUVECs in culture were harvested and mixed at a ratio of 4:1. Next, mixed iHepLPCs–HUVEC pellets were resuspended in a T&EM/EGM co-culture medium and transferred into a 24-well ultra-low attachment culture plate (Corning, Kennebunk, ME, United States) to form 3D cell spheroids for 24 h. Subsequently, after incubation for 24 h in a humidified incubator containing 5% CO_2_ at 37°C, the cell spheroids were collected and mixed with the neutralized type I collagen solution (final concentration, 1 mg/ml; BD Biosciences). This cell spheroids–collagen mixture (20–25 µl/scaffold) was later inoculated into pretreated scaffolds, keeping the number of iHepLPCs constant (400,000 cells/scaffolds). After gelation at 37°C for 1 h, the cell-loaded scaffolds were transferred into a 12-well plate. A T&EM/EGM co-culture medium was added gently to avoid disturbing the scaffolds. Monocultures of iHepLPCs (without HUVECs) under the same conditions with the same seeding density or monolayer cultures (2D culture) of iHepLPCs in Petri dishes served as the control.

To promote oxygen, nutrient, and waste exchange among the cells in the scaffolds, Rotary Cell Culture System^™^ (RCCS, Synthecon Incorporated, Houston, TX, United States) was used for the dynamic culture of the cells–scaffolds complex. First, 24 h after the seeding process of cell spheroids, the resultant cell-loaded scaffolds were gently transferred to culture vessels and filled with an HMM/EGM co-culture medium to initiate hepatic differentiation. The rotational speed was set at 15–20 rpm, and the culture medium was changed every other. The cell-loaded scaffolds were cultured for up to 3 weeks in the RCCS. To distinguish hepatocytes from endothelial cells within the co-cultures, CellTracker^™^ DiI and CellTracker^™^ DiO (Invitrogen, Carlsbad, CA, United States) were applied to label iHepLPCs and HUVECs, respectively. Briefly, the adherent cells at 80–90% confluence were incubated in a pre-warmed medium containing 25 μM CellTracker^™^ dye under growth conditions for 30–40 min. Finally, the dye-containing medium was replaced by a fresh medium, and the cells were incubated for another 30 min at 37°C. After washing with PBS solution, the labeled cells were harvested and inoculated for 7 days on the scaffolds, and then the scaffolds were harvested and captured using confocal laser scanning microscopy (CLSM, Wetzlar, Germany).

### Electron Microscopy

The harvested samples were prefixed in 2.5% glutaraldehyde solution (Solarbio) overnight at 4°C. Then, they were washed with PBS solution to remove the glutaraldehyde solution thoroughly and postfixed with 2% osmium tetraoxide solution (Solarbio) for at least 2 h at ambient temperature. For scanning electron microscopy (SEM) detection, the samples were lyophilized and sputter-coated with gold particles, and then images were captured using a JEOL-F200 microscope (Japan). For transmission electron microscopy (TEM) detection, after postfixation, the samples were embedded with Epon 812 to prepare ultrathin sections and visualized under a JEM-2000EX microscope (Japan).

### Cell Viability

Cell viability was assessed using a calcein-AM/EthD-1 staining kit (Invitrogen) according to the manufacturer’s instructions. First, the samples were collected at the indicated time points and washed with PBS solution. Then, they were incubated with a working solution of calcein-AM/EthD-1 for 2 h at 37°C. After washing with an FBS-free culture medium, they were loaded into a petri-dish with a glass bottom, and their images were captured using CLSM.

### Histology and Staining

#### Hematoxylin and Eosin (H&E) Staining

Before embedding in paraffin, the samples were harvested and fixed overnight in 4% paraformaldehyde (Solarbio). Paraffin sections (5 µm) were prepared at the Pathology Department at Dalian Medical University (Dalian, China). The sections were deparaffinized and stained with H&E solution as described previously (Wang et al., 2010), and images were captured using a Leica TCS microscope (Wetzlar).

#### Whole-Mount Immunofluorescence (IF) Staining

Whole-mount IF staining of the cell-loaded scaffolds was performed as described previously (Wang et al., 2010). In brief, the samples were collected at the indicated time points and fixed in 4% paraformaldehyde for 12 h. After thoroughly washing with PBS solution, the scaffolds underwent sequential membrane permeabilization and blocking with nonspecific antigens. Subsequently, they were incubated overnight at 4°C using the following primary antibodies: mouse anti-human ALB (dilution, 1:50; Santa Cruz Biotechnology, Santa Cruz, CA, United States), rabbit anti-human CYP3A4 (dilution, 1:100; Proteintech, China), rabbit anti-human MRP_2_ (dilution, 1:50; Proteintech), and Alexa Fluor^®^647 Mouse anti-Human CD31 (dilution, 1:100; BD Biosciences). Samples not incubated with specific primary antibodies served as negative controls. Next, cell-loaded scaffolds were incubated with FITC-conjugated Goat anti-Rabbit IgG Secondary Antibody or FITC-conjugated Donkey anti-Mouse Secondary Antibody (dilution, 1:200; Invitrogen) for 2 h at room temperature. DAPI (2 μg/ml, Research Organics, Cleveland, OH, United States) was used to counterstain the cell nucleus, and images were captured using CLSM.

#### Immunohistochemical (IHC) Staining

IHC staining was conducted as described previously (Liu et al., 2018). Briefly, deparaffinized sections were rehydrated and underwent an antigen retrieval process. Then, the samples were treated sequentially with 3% hydrogen peroxide and a nonimmune goat serum, followed by incubation with the same primary antibodies as those used in IF staining overnight at 4°C. Sections not incubated with specific primary antibodies served as negative controls. Following incubation with the primary antibodies, the samples were incubated again with a cocktail of biotinylated goat anti-rabbit or anti-mouse secondary antibodies for 15 min. Next, they were incubated with an avidin/biotin/peroxidase complex for 15 min. Following thorough washing, the sections were colored with a diaminobenzidine chromogenic solution, counterstained with hematoxylin, dehydrated, and mounted. Images were captured using a Leica TCS microscope. For IHC data quantification, six fields from a minimum of three samples were blindly analyzed using the NIH ImageJ plugin IHC Image Analysis Toolbox.

### Real-Time Quantitative PCR (RT-qPCR)

The total RNA of 2D cells and 3D cultures were extracted using TRIzol reagent (Ambion Life Technologies, Denmark) according to the manufacturer’s protocols. Then, 1 μg of RNA was reverse transcribed using a cDNA synthesis kit (TaKaRa Bio, Shiga, Japan). Real-time PCR was conducted using Agilent Technologies System (Peking, China) and SYBR^®^ Premix Ex Taq^™^II Kit (Takara Bio). Amplification was conducted for 40 cycles of 3 s each at 95°C, 30 s each at 58°C, and 30 s at 72°C. Gene expression was calculated using the 2^−ΔΔCt^ method and was normalized to that of β-actin. Each sample was analyzed in triplicate. The primer sequences are listed in Supplementary Table S2.

### Hepatic Function Detection

For the intake of acetylated low-density lipoprotein (ac-LDL), 3D constructs were incubated with 10 μg/ml DiI-ac-LDL (Invitrogen) for 5 h and then 1× Hoechst 33342 (Invitrogen) for another 1 h. After thoroughly rinsing with PBS solution, images were captured using CLSM.

The supernatants of 2D cultures, 3D monocultures, and 3D co-cultures were collected at the indicated time points to assess the secretion of human ALB. The amount of ALB secreted was determined using a Human Albumin ELISA kit (Bethyl Laboratory, Montgomery, TX, United States) according to the manufacturer’s instructions. The absorbance was measured using a Synergy NEO Hybrid Multi-Mode Microplate Reader (BioTek, VT, United States). To determine urea production, 2D cultures, 3D monocultures, and co-cultures were incubated in a medium containing 3 mM NH_4_Cl (Solarbio) for 24 h, after which the supernatants were collected. The urea concentration was measured using a QuantiChromTM urea assay kit (BioAssy Systems, Hayward, CA, United States) according to the manufacturer’s protocols. The total DNA of each group was quantified using the Pico Green DNA assay kit (Gibco), and all data were corrected for culture time and total DNA content.

Subsequently, the 3D constructs were incubated for 1 h with 1× Hoechst 33342 and another 30 min with 10 µg/ml 5(6)-carboxy-2′, 7′-dichlorofluorescein diacetate (CDCFDA, Invitrogen) to determine functional polarization. They were washed with ice-cold PBS containing calcium and magnesium and imaged using CLSM.

Alternatively, 2D differentiated cells and 3D cultures were incubated with 25 μM rifampicin (induction of CYP3A4) or 50 μM omeprazole (induction of CYP1A2) (all from Sigma-Aldrich) dissolved in a culture medium for 72 h to evaluate CYP450 induction. The relative gene expression was quantified using RT-qPCR as described above.

### *In Vivo* Subcutaneous Implantation in Mice

The Animal Care and Use Committee of Dalian Medical University approved all procedures. The experiments were conducted under animal care protocols. All animals used in this study were 6–8-week-old male BALB/C nude mice weighing 22–25 g (Liaoning Changsheng Biotechnology Co., Ltd., China). The mice were randomly assigned to two groups with three time points (7, 14, and 21 days). Then, 24 h after cell spheroid inoculation, cell-loaded scaffolds (diameter: 6 mm; thickness: 2 mm) were preincubated in a serum-free medium for 2 h. The cells were subcutaneously implanted in the lateral subcutaneous pocket of each mouse under general anesthesia using 4% chloral hydrate (10 µl/g of body weight of mice). Acellular scaffolds served as the blank control. The healing process at the incision region was monitored daily, and no deaths were registered during the experiment. Finally, the mice were euthanized at the indicated time points to check for the implantation outcomes, after which the samples were collected along with the surrounding tissue for histological examination.

### Statistical Analyses

All quantitative experiments were conducted in triplicate, and the results were expressed as mean ± standard deviation. All statistical analyses were performed using GraphPad Prism 8. First, the two-tailed unpaired *t-test* was performed to compare the statistical significance between the two groups. Then, a two-way analysis of variance was performed with Tukey correction for multiple comparisons of multiple values. A *p*-value of <0.05 was considered statistically significant.

## Results

### Morphology of the Composite SFC Scaffolds

**FIGURE 1:**
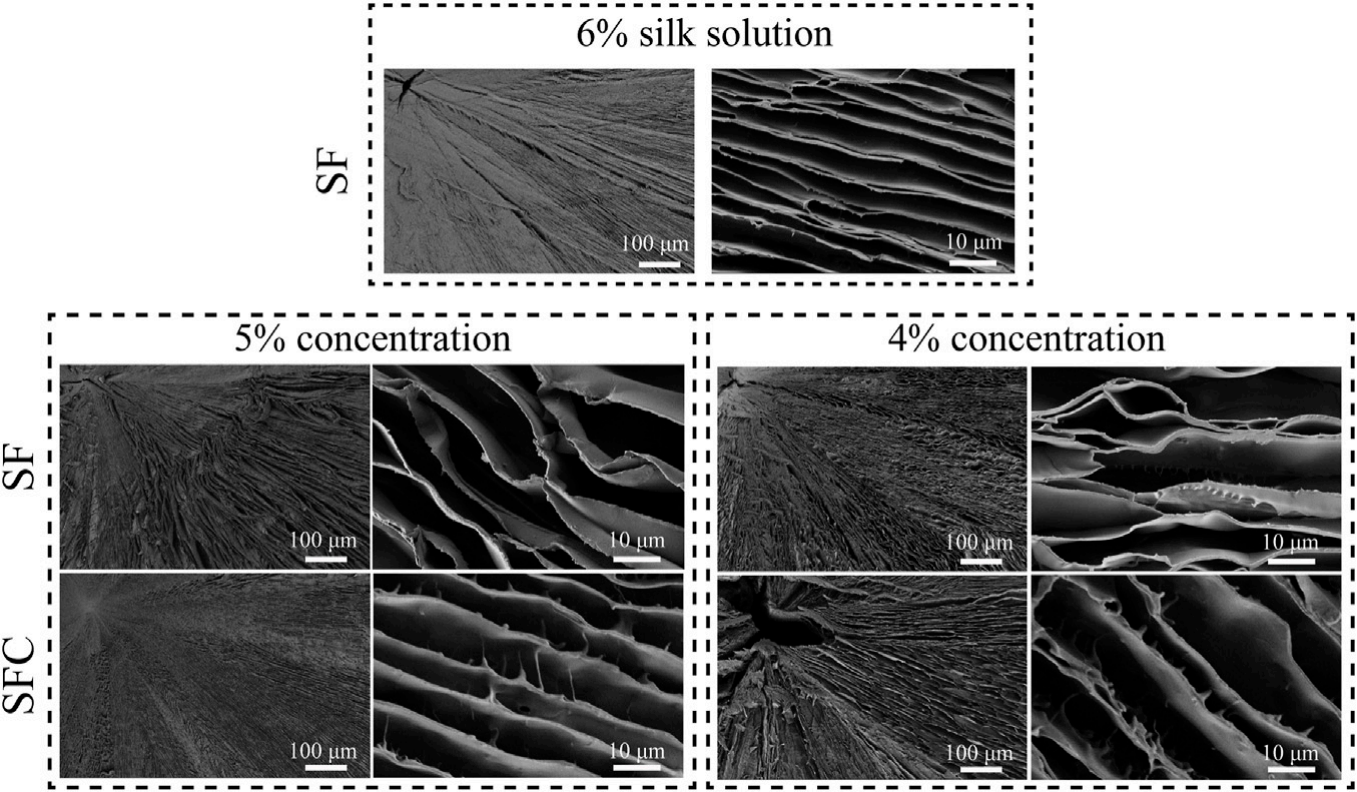
SEM images of SF and SFC scaffolds at different concentrations (w/v).

Biomimetic hepatic lobule-like scaffolds fabricated from various solutions (4–6% SF, and 4–5% SFC) were characterized based on their morphology using SEM (Figure 1). Although the scaffolds exhibited radial pores formed by aligned lamellar sheets, the gap distances increased with decreasing SF concentrations. Specifically, the 6 and 5% SF concentrations ensured the formation of well-defined radially aligned lamellar sheets. Locally, a pore collapse could be seen when the concentration of SF was reduced by 4% (Supplementary Figure S2). The lamellar sheets of SFC scaffolds were relatively rough, with projections on the wall surface compared with SF scaffolds, which might promote cell adhesion and migration.

### Physical Performance of the Composite SFC Scaffolds

#### Structural and Degradation Properties of the Composite SFC Scaffolds

**FIGURE 2:**
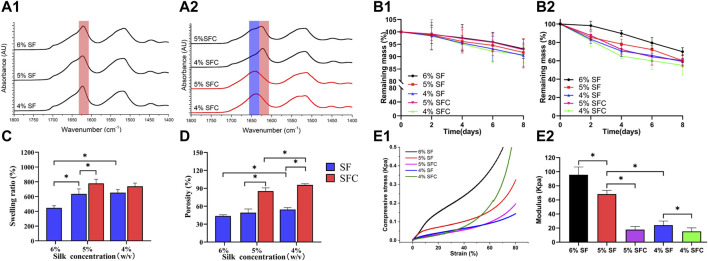
Physical performance of SF and SFC scaffolds at concentrations of 4, 5, and 6% (w/v). FTIR spectra of SF **(A1)** and SFC **(A2)** scaffolds at different concentrations (w/v) before (red line) and after (dark line) autoclaving. Time-dependent weight change of SF and SFC scaffolds in a PBS buffer **(B1)** and protease XIV solution **(B2)** are shown. Adding collagen I increased the swelling ratio **(C)** and porosity **(D)**. A compressive stress–strain curve showing SF and SFC scaffolds in the hydrated state **(E1)** and the summary of the compressive modulus for SF and SFC scaffolds **(E2)**. (*p < 0.05).

The prominent SFC scaffold peaks at different concentrations were seen at approximately 1630 cm^−1^, which were characteristic peaks assigned to β-sheets (1616–1637 cm^−1^ in the amide I region) and were similar to that of the SF scaffolds after autoclaving (Figure 2A). Therefore, collagen I rarely affected SF structural transitions from random coils to β-sheets, causing water insolubility and stability to the freeze-dried hepatic lobule-like silk scaffolds. Immersing the SF and SFC scaffolds in PBS solution resulted in a slow degradation (Figure 2 B1). The weight of the scaffolds was reduced faster by immersing them in protease XIV solutions. Furthermore, after 8 days of incubation, 60.3 ± 2.2% and 55.0 ± 10.4% of the original weights remained for the 5 and 4% SFC groups, respectively. In contrast, the original mass of the 5 and 4% SF scaffolds was reduced to 60.2 ± 6.7% and 59.1 ± 3.8% at similar incubation periods (Figure 2 B2). Thus, adding collagen had little effect on the degradation rate of the scaffolds, which ensures long-term support for the growth and differentiation of *in vitro* cells.

#### Permeability and Mechanical Properties of the Composite SFC Scaffolds

Swelling ratio and porosity are crucial for the exchange of nutrients and waste throughout the scaffold as they are the main factors that affect permeability (Huang et al., 2019). The swelling ratio of the 5 and 4% SFC scaffolds was 778.1 ± 56.5% and 738.4 ± 44.3% respectively, which were significantly higher than those of the SF scaffolds (5%: 635.0 ± 68.3%; 4%: 653.5 ± 41.4%) (*p* < 0.05, Figure 2C). This increase was also related to the increased porosity. Specifically, the scaffold porosity of 5% SFC (85.4 ± 5.4%) and 4% SFC (95.7 ± 2.1%) was higher than that of 5% SF (49.3 ± 6.0%) and 4% SF (54.5 ± 3.1%) (*p* < 0.05, Figure 2D). Figure 2E1 showed the compressive stress–strain curves for the hepatic lobule-like silk scaffolds fabricated from various solutions. The compressive modulus decreased gradually with a lower concentration of SF, which is consistent with the results in our previous experiments (Figure 2E2). The compressive modulus of the 5% and 4% SF scaffolds were 68.2 ± 5.5 kPa and 24.2 ± 5.8 kPa, respectively, which were significantly higher than those of the 5% SFC (17.7 ± 4.4 kPa, *p* < 0.05) and 4% SFC (15.3 ± 4.9 kPa, *p* < 0.05) scaffolds. Therefore, while the mechanical properties of the collagen I-modified scaffolds were optimized, the modulus was closer to that of the native liver tissue (approximately 2.8–7.4 kPa).

Taking into account the scaffold integrity, improvement in physical properties (swelling ratio, porosity, and mechanical properties), and long-term hepatic culture (degradation properties) *in vitro* and *in vivo*, 5% SFC scaffolds were adopted in the following experiments, and 5% SF scaffolds served as the control.

### C3A Distribution and Performance on the Composite SFC Scaffolds

**FIGURE 3:**
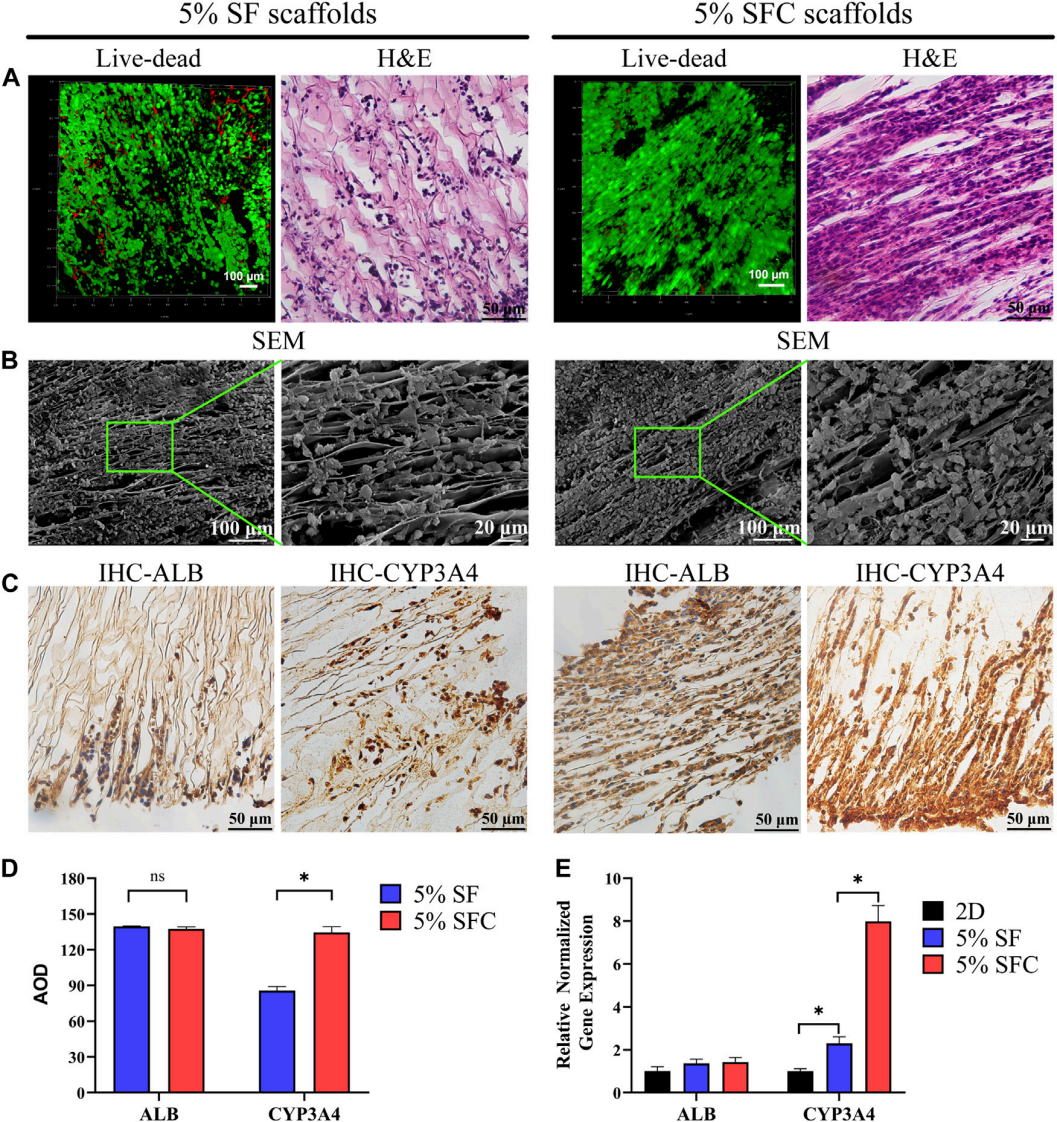
Biocompatibility of 5% composite SFC scaffolds (w/v). The viability staining and H&E staining of cells in both scaffolds **(A)**. SEM images **(B)** showing the cell growth pattern on the surface of both scaffolds. IHC detection **(C)** of ALB and CYP3A4 expression of C3A cells in both scaffolds. Quantitative analysis of IHC staining **(D)** and functional gene expression of C3A cells in both scaffolds **(E)**. (*p < 0.05).

The majority of cells in both 5% SFC and 5% SF scaffolds exhibited good viability after 2 weeks of culture (Figure 3A). H&E staining revealed a more detailed growth pattern within 5% SF scaffolds, wherein C3A cells migrated to and were distributed in the margin, while cell migration in 5% SFC scaffolds was significantly promoted and formed a bionic hepatic cord-like configuration (Figure 3A). Notably, cells were in closer contact with each other within 5% SFC scaffolds. On the scaffold surface, C3A cells adhered to laminar sheets while some cells resided along with the spacing between both scaffolds as showed by SEM (Figure 3B). This finding, combined with H&E results, further indicated that more cells had migrated from the surface into the interior of the 5% SFC scaffold.

No significant differences were detected in the ALB expression between the two groups. However, C3A cells cultured in 5% SFC scaffolds showed significant upregulation in CYP3A4 expression in both the protein and gene levels (*p* < 0.05, Figures 3C-E) compared with cells cultured in 5% SF scaffolds. This suggested that the 5% SFC scaffolds were superior to the SF scaffolds in promoting the expressions of mature hepatic markers.

The above results indicated that 5% SFC scaffolds were equipped with not only improved swelling ratio/porosity and ameliorative mechanical properties but also the ability to promote hepatocytes migration and function. Thus, 5% SFC scaffolds were chosen to serve as a more suitable template to construct a biomimetic hepatic lobule-like compartment.

### Growth Profile of Cells on the Composite SFC Scaffolds

**FIGURE 4:**
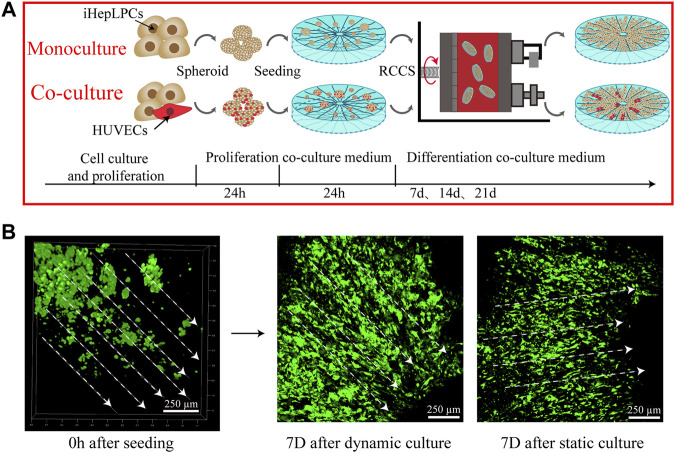
A schematic showing the construction process of hepatic lobule-like cultures **(A)**. Distribution and morphological changes in iHepLPCs (GFP-transfected) after dynamic or static culture conditions **(B)** are shown.

The construction process of the 3D hepatic lobule-like cultures is briefly shown in Figure 4A. Cellular spheroids composed of iHepLPCs and HUVECs with a diameter of 50–200 µm were prepared to improve the inoculation efficiency and cell viability (Supplementary Figure S3). After 1 week of cell seeding, we observed that the cells in the SFC scaffolds were distributed evenly throughout the scaffold, primarily as single cells or small aggregates. Most of the iHepLPCs (GFP-transfected) were localized along the radial laminar sheet and channels, displaying a well-organized directional configuration. Compared with the static culture, iHepLPCs (GFP-transfected) cultured in RCCS were considered more stretched and complete in shape (Figure 4B).

**FIGURE 5:**
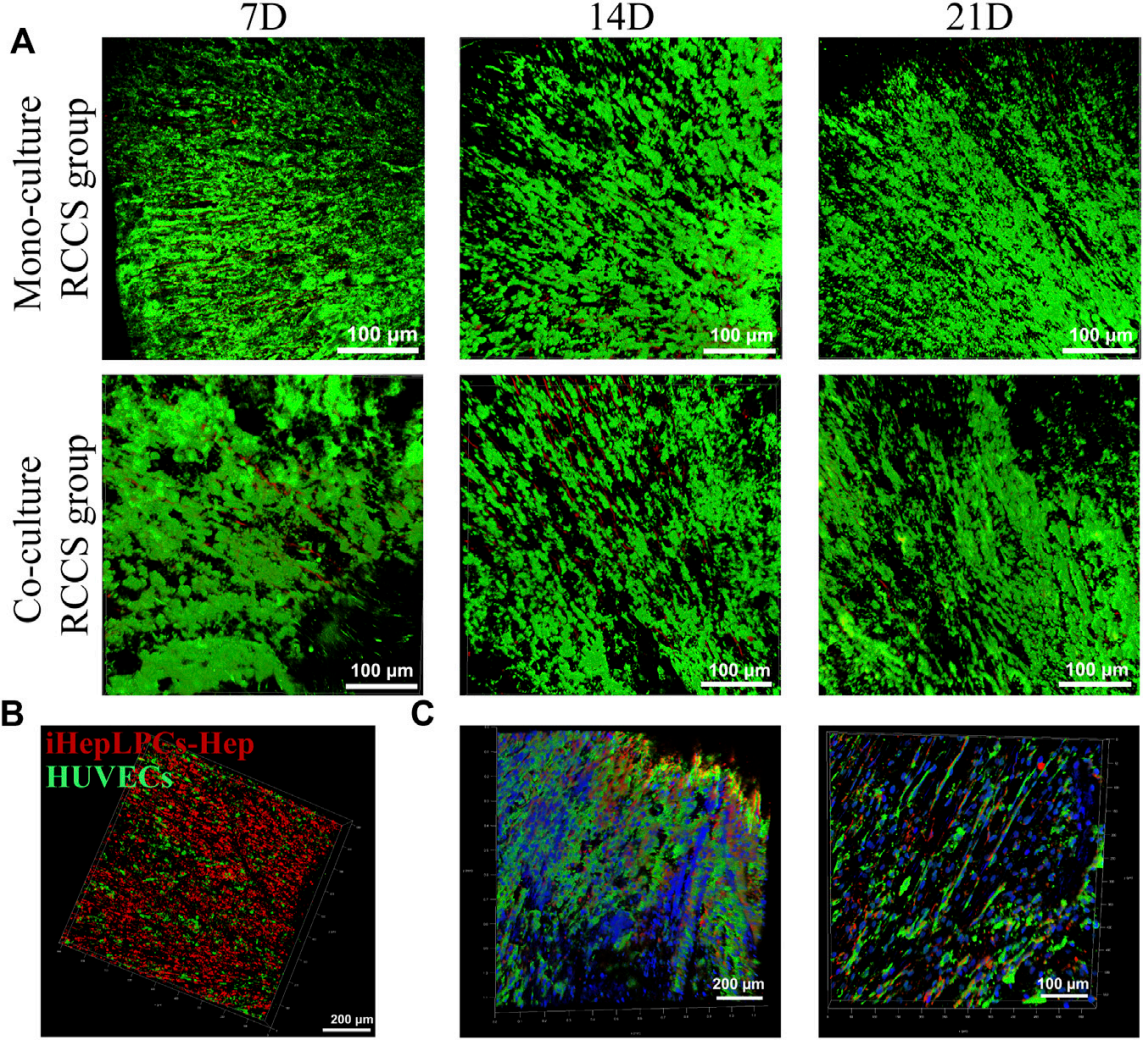
Growth profiles of iHepLPCs on composite SFC scaffolds. The viability staining of both monocultures and co-cultures is shown. Aligned growth pattern of the monocultured or co-cultured cells over time were observed using CLSM **(A**). Radially aligned hepatocyte and endothelial cell interphase arrangement of 3D co-cultures was revealed using cell-tracker dye staining **(B)** (red, iHepLPC-Heps; green, HUVECs) and IF staining **(C)** (green, ALB-FITC-conjugated secondary antibody-labeled iHepLPC-Heps; red, CD31-Alexa Fluor647-labeled HUVECs; blue, DAPI) under CLSM.

After 3 weeks of culture in RCCS, iHepLPCs and HUVECs displayed good viability, evidenced by their remarkably bright green fluorescence and inconspicuous red fluorescence under CLSM (Figure 5A). This result suggested that the SFC scaffolds provided a suitable microenvironment for the *in vitro* culture of hepatocytes and stromal cells. However, the number of cells barely increased over time, as evidenced by the unvaried green fluorescence. This indicates that iHepLPCs lost their proliferative stem cell properties and initiated hepatic differentiation in the 3D-culture environment.

A locally interphase arrangement pattern of iHepLPCs (labeled with DiI, red) and HUVECs (labeled with DiO, green) was observed in 3D co-cultures (Figure 5B). This distribution profile was further confirmed by IF staining after 14 days of dynamic culture (Figure 5C). Moreover, attributed to a higher initial inoculation radio (4:1), a larger number of hepatocytes than HUVECs were observed.

### Cell Morphology and Organization on the Composite SFC Scaffolds

**FIGURE 6:**
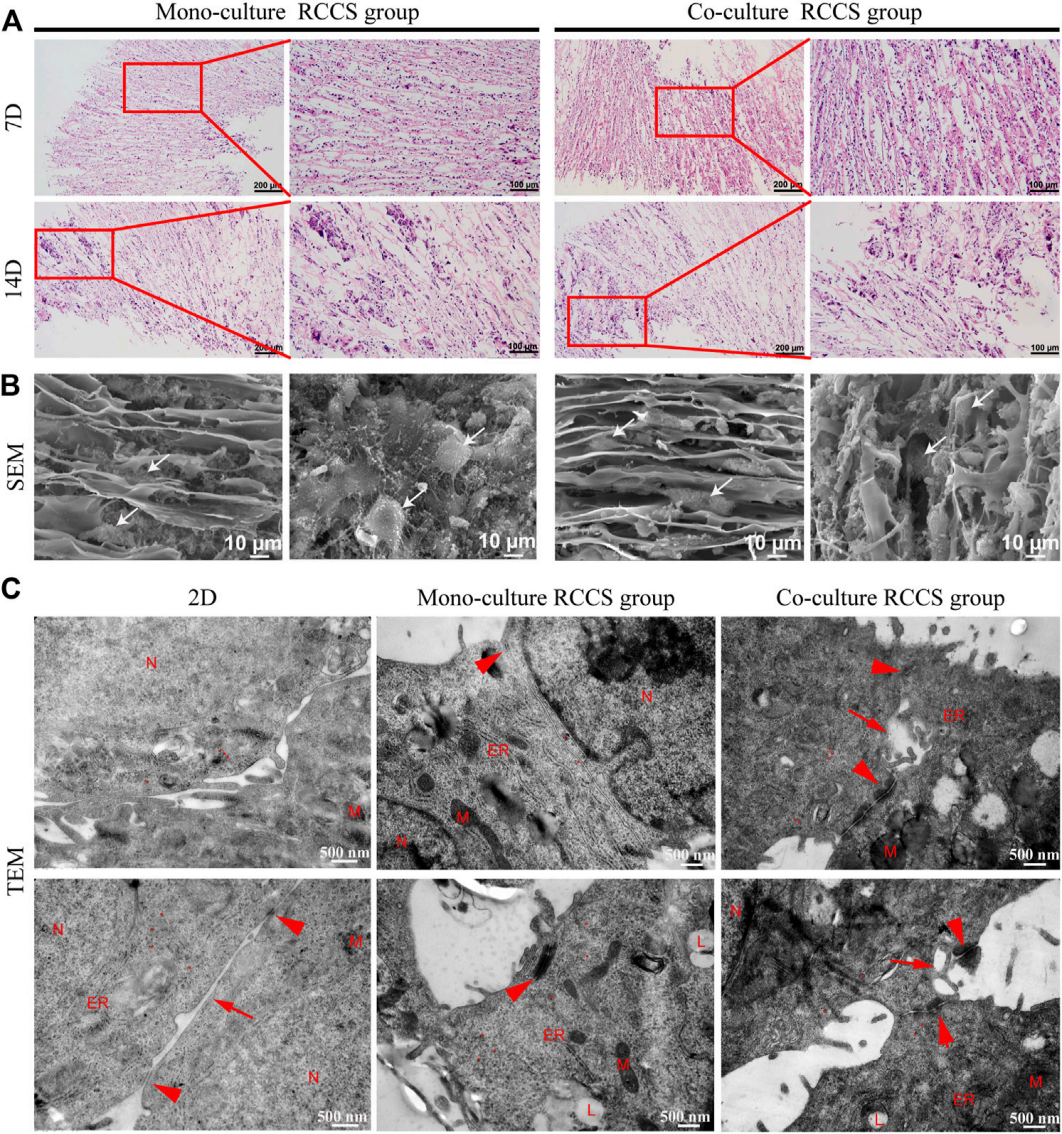
Morphological characteristics of cells on composite SFC scaffolds. H&E staining **(A)** showed that cells displayed a compact but radially organized morphology. Locally clustered cells were observed after 14 days of a dynamic culture. SEM images **(B)** showed the cell growth pattern on the surface of composite SFC scaffolds. Cells (white arrows) exhibited long spindle or polygon shapes and migrated along with the radial pores of scaffolds. Ultramicroscopic TEM images **(C)** indicated the improved maturation of iHepLPC-Heps after 3D dynamic culturing, especially more cell junctions (red arrowheads) and bile canaliculi-like structures (red arrows) were formed in co-cultured iHepLPC-Heps. (Nucleus- N, mitochondria- M, endoplasmic reticulum- ER, lipid droplets- L, red dot-glycogen).

H&E staining displayed that the cells were located between the spaces of laminar sheets, displaying a compact but radial-organized morphology after 7 days of culture. Most cells were large, with round nuclei and eosinophilic cytoplasm. After 14 days, local clustering of the cells could be observed, accompanied by disruption of the structural integrity on the scaffold (Figure 6A). This indicated that the scaffolds were degrading and replaced by cell-secreted ECM. SEM further revealed the cell distribution on the scaffold surfaces (Figure 6B). Although the cells were grown with the directional laminar sheets in a long spindle or polygonal shape with rich ECM secretion, the diverse morphology of the iHepLPCs during maturation led to an indistinguishable morphology of the two cell types.

Subsequently, the ultramicroscopic morphology of the iHepLPC-Heps on SFC scaffolds was characterized using TEM (Figure 6C). More ultrastructural characteristics of mature hepatocytes (increased number of mitochondria, endoplasmic reticulum, and appearance of lipid droplets) were displayed in iHepLPC-Heps after 14 days of 3D dynamic culture than in 2D differentiated cells. More importantly, compared with the monoculture RCCS group, co-cultured iHepLPC-Heps exhibited more cell junctions and bile capillary-like structures, indicating improved maturation. This finding suggested that the unique microenvironment, involving both radial scaffold architecture and endothelial cells, promoted the maturation of iHepLPCs.

### Functional Gene and Protein Expression Profile of iHepLPC-Heps on the Composite SFC Scaffolds

**FIGURE 7:**
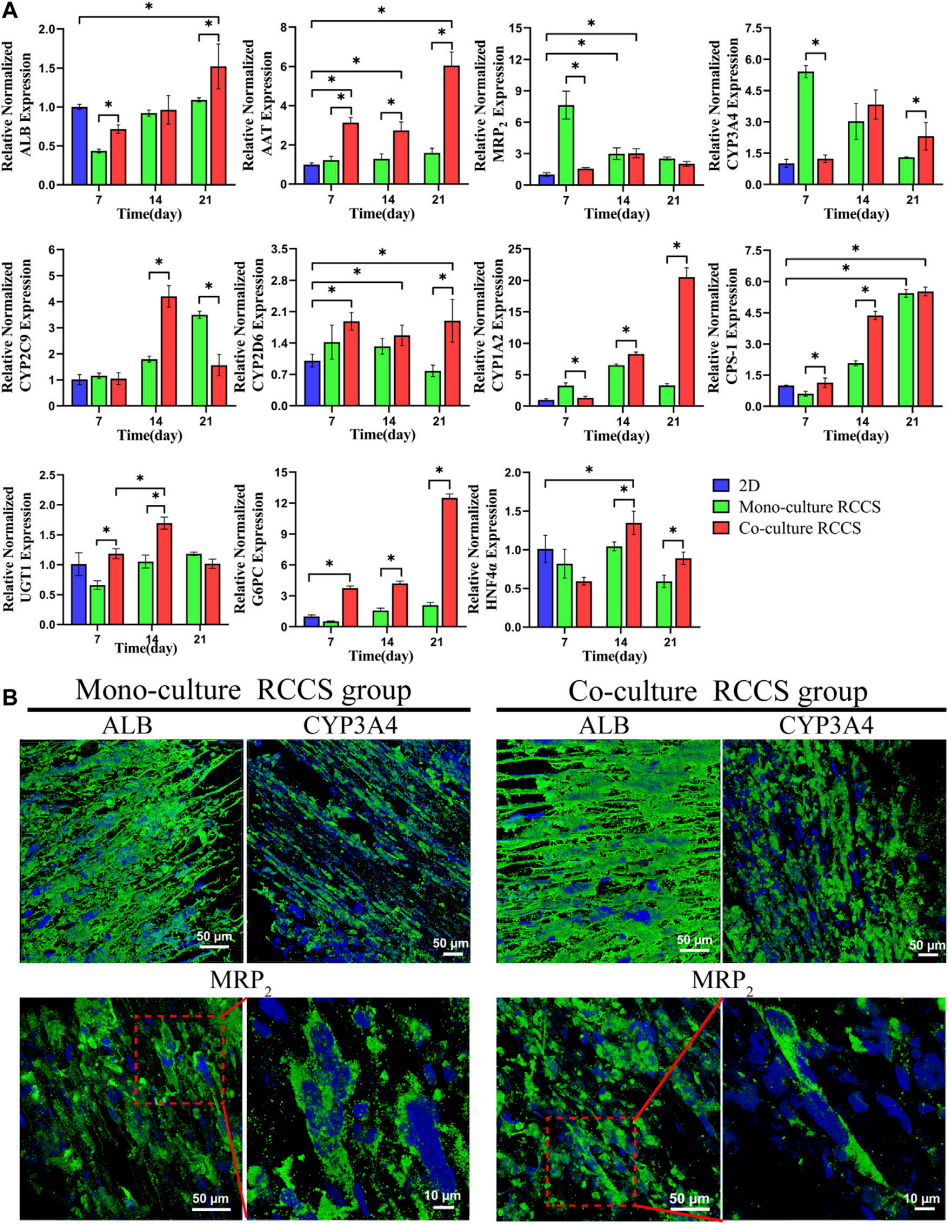
Functional gene and protein expression in different culture conditions using RT-qPCR **(A)** and IF staining **(B)**. Higher functional gene expression was found in the 3D co-cultures than in the 2D cultures and 3D monocultures **(A)**. iHepLPC-Heps in both groups expressed ALB, CYP3A4, and MRP_2_ after 14 days of culture **(B)**. (*p < 0.05).

The transcriptional expression levels of some critical genes for functional hepatocyte differentiation were detected using RT-qPCR, including those encoding secretory proteins (ALB and AAT), multidrug resistance-associated protein 2 (MRP_2_) transporters, vital cytochrome P450 (CYP3A4, CYP1A2, CYP2C9, and CYP2D6), some metabolism-related enzymes (CPS-1, UGT1, and G6PC), and HNF4α (Figure 7A). Compared with 2D differentiated iHepLPC-Heps, the 3D dynamic cultures exhibited significantly improved transcript expression levels of the genes. Furthermore, a higher expression level with most functional genes was observed in the 3D co-cultured iHepLPC-Heps than in the 3D monocultures. This suggests the importance of endothelial cells in promoting the expression of hepatocyte functional gene *in vitro*. Notably, the expression levels of most genes gradually increased over time, which might benefit their long-term application in a bioartificial liver support system or drug screening test. IF staining results showed that both groups expressed ALB, CYP3A4, and MRP_2_ after 14 days of dynamic culture, although no significant difference was detected, which was consistent with the corresponding gene expression levels (Figure 7B).

### Functional Evaluation of iHepLPC-Heps on the Composite SFC Scaffolds

**FIGURE 8:**
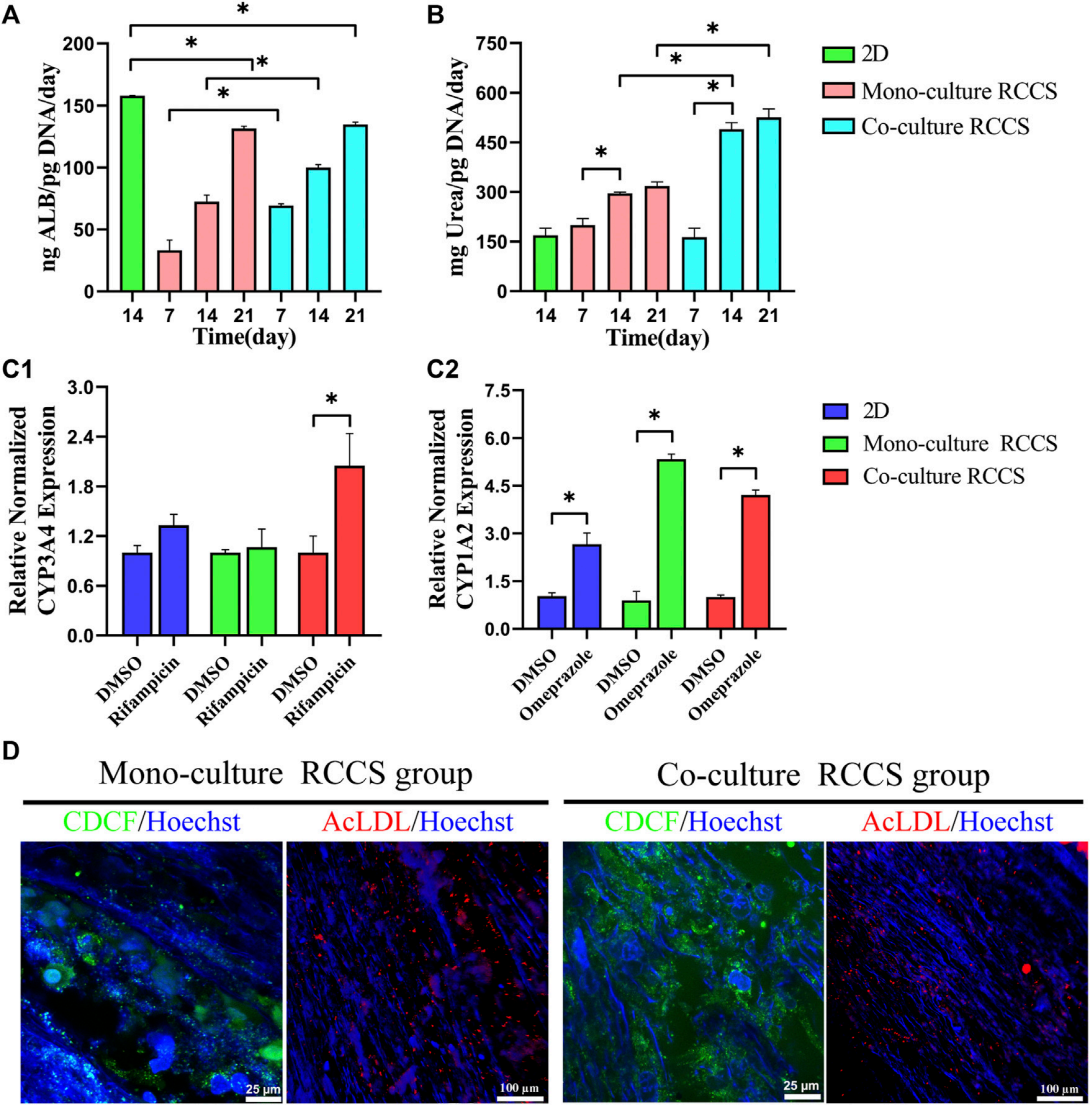
Functional evaluation of iHepLPC-Heps on composite SFC scaffolds. Albumin secretion of different hepatic cultures was assayed using ELISA **(A)**. Urea synthesis of iHepLPC-Heps cultured under different conditions **(B)**. Induction of CYP3A4 **(C1)** and CYP1A2 **(C2)** expression in response to stimulation with omeprazole and rifampicin, assayed using RT-qPCR. DiI-LDL uptake and CDCFDA staining **(D)** of iHepLPC-Heps were examined in both groups. (*p < 0.05).

The albumin production and urea synthesis in the 3D constructs increased progressively over 3 weeks of culture. Albumin secretion in the co-culture group was significantly higher than that in the 3D monocultures within the first 2 weeks (Figure 8A). Compared with the 2D differentiated iHepLPC-Heps, the 3D cultures on the composite SFC scaffolds exhibited elevated levels of urea production, especially co-cultured hepatocytes (Figure 8B).

Omeprazole treatment upregulated CYP1A2 expression by 2-fold, 4-fold, and 1-fold in the co-cultures, monocultures, and 2D differentiated cells, respectively (over DMSO-treated controls, *p* < 0.05). Although rifampicin treatment upregulated the expression of CYP3A4 by 1.8-fold in the co-cultures (*p* < 0.05), a small but insignificant upregulation was observed in mono-/2D cultures (over DMSO-treated controls, Figure 8C). This finding suggested the role of stromal cells in the enhanced and stable promotion of CYP responses to drugs.

Additionally, more polarized epithelial cells in 3D co-cultures with bile canaliculi-like structures were observed using CDCFDA staining, as evidenced by the increased amount of peri-nuclearly clustered fluorescein instead of diffusely distributed fluorescein in the cytoplasm. Although no evident difference was observed, LDL uptake assays showed that 3D cultures could take up LDL (Figure 8D). Collectively, these data revealed that iHepLPCs that differentiated in composite SFC scaffolds exhibited functionally mature hepatocyte-like characteristics. Moreover, endothelial cells could promote and maintain most functions of hepatocytes in direct co-culture.

### *In Vivo* Compatibility of Cell–Scaffold Complex

**FIGURE 9:**
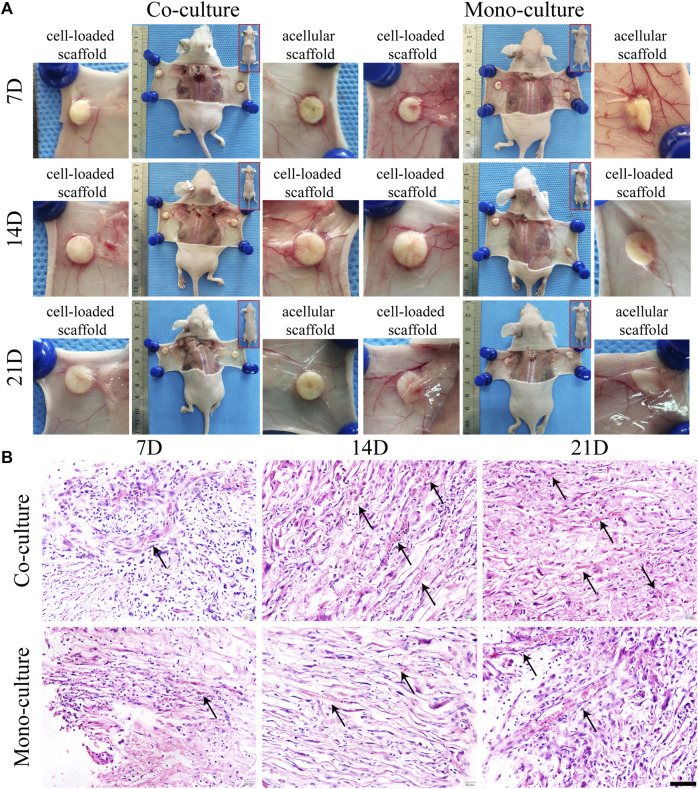
Morphological characterization of cell-loaded cultures after transplantation. General growth morphology of scaffolds in each group was shown at 7, 14, and 21 days after transplantation **(A)**. H&E staining **(B)** showed radially distributed cells in the scaffolds. Host blood vessels (black arrows) grew abundantly within the pores of all cultures, especially the co-cultures. Scale bar, 50 µm.

The morphology and function of the cell–scaffold complex *in vivo* were evaluated. Both monocultures and co-cultures were subcutaneously transplanted into nude mice and acellular SFC scaffolds were used as control. Macroscopically, the incisions healed completely within 1 week without any signs of infection, reddening, or festering. All the implants were complete without degradation and were enveloped by a transparent membrane-like tissue. No evident difference was observed between the cell-loaded and acellular scaffolds. Nevertheless, visible blood vessels extending toward the graft were found, where a dense vascular network was generated after 7 days of implantation. This network is thought to maintain nutrient supply for the epithelial layer of the grafts (Figure 9A).

H&E staining results displayed that the cells on the scaffolds were radially organized between the spaces of laminar sheets with large round nuclei and eosinophilic cytoplasm, consistent with the *in vitro* results (Figure 9B). Notably, 3D complexes exhibited a biomimetic architecture, with host capillaries radially extending in the pores of SFC scaffolds and aligning to hepatocytes. There were more infiltrated or neovascular capillaries in the co-culture group than that in the monoculture group. However, we failed to detect the integration of human HUVECs in mice capillaries. Nevertheless, our finding showed that endothelial cells were critical in activating angiogenic signaling to promote neovasculogenesis rather than functionally integrating into host capillaries.

**FIGURE 10:**
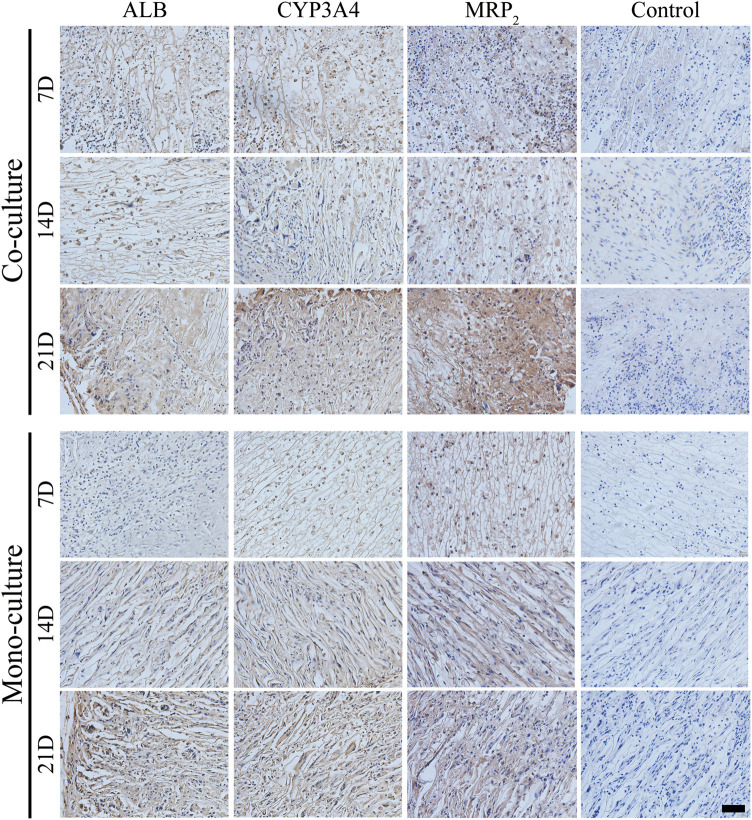
IHC detection of ALB, CYP3A4, and MRP_2_ expression in both groups after transplantation. Protein expressions increased gradually over time. Compared with monocultures, the co-culture group showed upregulated expressions at each time point. Scale bar, 50 µm.

Subsequently, the maturity level of the differentiated iHepLPCs was characterized by evaluating the expression of ALB, CYP3A4, and MRP_2_ using IHC staining. The expression of ALB, CYP3A4, and MRP_2_ increased gradually in both groups with a prolonged transplantation time, showing spontaneous cell differentiation toward iHepLPC-Heps (Figure 10). Furthermore, co-cultures had significantly higher ALB, CYP3A4, and MRP_2_ expression than the monocultures at all-time points, consistent with the *in vitro* results.

## Discussion

Recently, increasing evidence pinpoints the importance of matching the performance of biological scaffolds with the *in vivo* parameters of the microenvironment for engineering structural and functionally equivalent hepatic tissue constructs *in vitro* (Luo et al., 2021; Stoppato et al., 2015). Physical properties, such as porosity, water uptake ability, and mechanical properties, are important features of SF porous scaffolds that modulate the biological behavior of cells toward the desired engineered tissue (Kundu et al., 2013), especially the liver, due to its intricate architecture and specific functionality. Previously, we constructed a radially aligned porous SF scaffold and demonstrated its feasibility in the construction of engineered liver lobule-like tissue. However, unsatisfactory physical properties and lack of integrin-mediated cell-binding sites limited its applications. Therefore, we enhanced the physical properties of the scaffold and optimized the construction of 3D-engineered liver models based on the previous model as follows.

First, integrin-mediated cell-binding sites were introduced into the SFC composite hepatic lobule-like scaffolds by incorporating collagen I. Integrin signaling is one of the major factors that regulates cell attachment and initiates downstream signaling for hepatic cellular responses, such as cell survival, proliferation, and cellular functions (Greenhalgh et al., 2019; Weng et al., 2020). Upregulated C3A cell attachment and migration and CYP3A4 expression demonstrated the importance of adding active integrin sites to the scaffold. Moreover, SFC scaffolds displayed better optimized porosity, swelling ratio, and mechanical properties compared to SF scaffolds. Other studies have confirmed the effective improvement of physical performance of SFC hydrogel or electrospun fibers (Buitrago et al., 2018; Maghdouri-White et al., 2014), whereas this is the first study to fabricate SFC porous scaffolds by directional lyophilization. The improvement may be attributed to the increased viscosity of the SF solution when mixed with an unneutralized acidic collagen solution, which altered ice crystal formation (Cho et al., 2012). Therefore, SFC composite scaffolds provide a new strategy for fabricating a biomimetic liver tissue that structurally and physiologically replicates the human native liver tissue *in vitro*.

Second, primary human hepatocyte (PHH) derived functional liver cells were adopted to facilitate downstream applications for seed cells as they are a prerequisite and key to the success of constructing functional engineered liver tissue. Deficiency in certain liver functions or potential tumorigenicity of hepatocarcinoma cell lines or stem cell-derived hepatocytes is currently one of the major reasons that hinders the application of tissue-engineered livers in drug screening and alternative therapy (Bhatia et al., 2014). However, iHepLPCs converted from PHH could efficiently revert to the mature hepatic state and exhibit enhanced liver-specific functions close to native liver in 3D spheroids culture (Fu et al., 2019; Liu et al., 2021). Moreover, good biocompatibility and the potential for individualized treatment could further be beneficial to the incorporation of functional liver lobule-like tissue we constructed using iHepLPCs into the bioartificial liver to individually benefit patients with liver failure or for the short/long-term hepatotoxicity screening of drugs.

Finally, we used the RCCS bioreactor culture system to dynamically incubate 3D cultures; a more stretched and intact cell morphology was observed and long-term viability was achieved after dynamic culture. Notably, the dynamic nature of the culture system, possessing microgravity, low shear force, and high exchange efficiency of O_2_, nutrients, and waste, partly overcomes the shortcomings of a static culture (Li et al., 2009). Studies have demonstrated that long-term culture and promotion of cell viability and proliferation can be achieved using this system (Ferrarini et al., 2013; Zheng et al., 2012). Moreover, especially for hepatocytes cultured *in vitro*, sufficient oxygen supplementation and mass transportation are vital. These results provide the guarantee for applying the hepatic lobule-like tissue as a cell block in biological artificial liver.

Based on the strong influence of endothelial cells on the fate of hepatocytes *in vivo* and *in vitro* (Cuvellier et al., 2021; Ding et al., 2010), we used HUVECs as co-cultured nonparenchymal cells. Compared with the monoculture group, HUVECs promoted maturity of hepatocytes. This finding was consistent with that of our previous work and other studies (Kim et al., 2017; Wang et al., 2022; Ware et al., 2018). However, the mechanisms underlying the positive effect of HUVECs have not been fully elucidated. Besides direct contact, paracrine cytokines secreted by HUVECs have been reported to be involved in hepatocyte function. For example, glial cell line-derived neurotrophic factor was found to activate downstream pathways through the phosphorylation of MET to promote liver functions (Liu et al., 2021). In addition, vascular endothelial growth factor was found to activate PI3K/AKT pathways to promote hepatocyte maturity (Kim et al., 2021; LeCouter, 2003).

A subcutaneous transplantation experiment was performed using nude mice to verify the biocompatibility and biofunctions of hepatic lobule-like cultures *in vivo*. Consistent with the *in vitro* results, the cells migrated within the scaffold. The scaffolds’ vascularization occurred rapidly, ensuring the survival of hepatocytes and their further differentiation. The radial pores of the scaffold provided space and guidance for the growth of host blood vessels, forming a highly bionic sinusoid-hepatic plate arrangement with adjacent hepatocytes. Moreover, endothelial cells in the co-culture group resulted in an increased number of host vessels and an expanded distribution range, indicating that HUVECs may play a role in the induction of angiogenesis. Jung, H. R. et al. subcutaneously implanted HUVEC–Huh7 cell spheroids into nude mice. Compared with monocultured 3D Huh7 spheroids, HUVECs induced the formation and maturation of new vessels in Huh7 spheroids (Jung et al., 2017). However, based on our extensive literature search, very few studies have reported the angiogenesis effect of HUVECs *in vivo* based on SF porous scaffolds. Therefore, this study provided a strategy for vascularizing SF porous scaffolds *in vivo* based on 3D-constructed tissue transplantation.

Although the influence of scaffold microarchitecture on cell biological behavior has been demonstrated in various culture models, the exact mechanism is unknown. This is mainly due to the lack of effectively paired scaffolds, wherein one scaffold has highly arranged configurations and the other one does not. Meanwhile, these paired scaffolds should have identical physical properties, such as mechanics or porosity, which are essential because increasing evidence indicates their strong impact on cell behavior. The versatile plasticity and tunable properties of SF favor the fabrication of adaptably matched scaffolds to explore the underlying mechanism. Therefore, SFC composite lobule-like scaffolds with radial configuration and optimized physical performances provide a basis for understanding how the microstructure affects hepatocyte behavior.

## Conclusion

A bioengineered biomimetic hepatic lobule-like culture was constructed by co-culturing human hepatocytes and endothelial cells on composite SFC scaffolds fabricated by directional freezing and lyophilization. This composite SFC scaffold featured radially aligned lamellar sheets, with physical performances similar to those of the normal liver matrix and with better biocompatibility. The dynamically co-cultured human hepatocytes and endothelial cells in the scaffolds exhibited a biomimetic radially organized interphase arrangement pattern and well-maintained functionality compared with the monoculture *in vitro* and *in vivo*. Thus, this culture system can provide a platform for drug industries or for constructing a functional bioartificial liver unit.

## Data Availability

The original contributions presented in the study are included in the article/Supplementary Files, further inquiries can be directed to the corresponding author.
