# Supplementary material for: Development of Biomimetic Hepatic Lobule-Like Constructs on Silk-Collagen Composite Scaffolds for Liver Tissue Engineering
Source: Front Bioeng Biotechnol. 2022 Jun 23;10:940634. doi: 10.3389/fbioe.2022.940634 (PMC9260023; doi:10.3389/fbioe.2022.940634)
Supplement: Supplementary file 1 [file DataSheet1.docx]

Supplementary Material


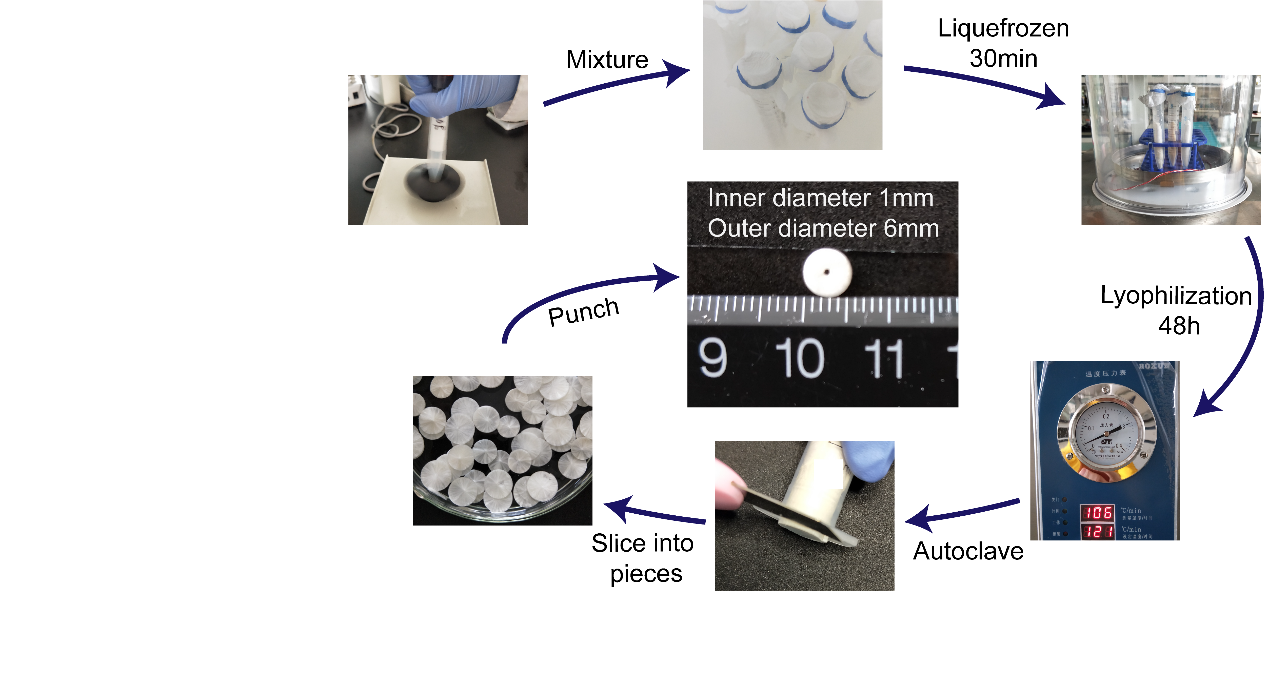


**Supplementary Figure 1**. Fabrication process of SFC composite biomimetic radially aligned porous scaffolds.


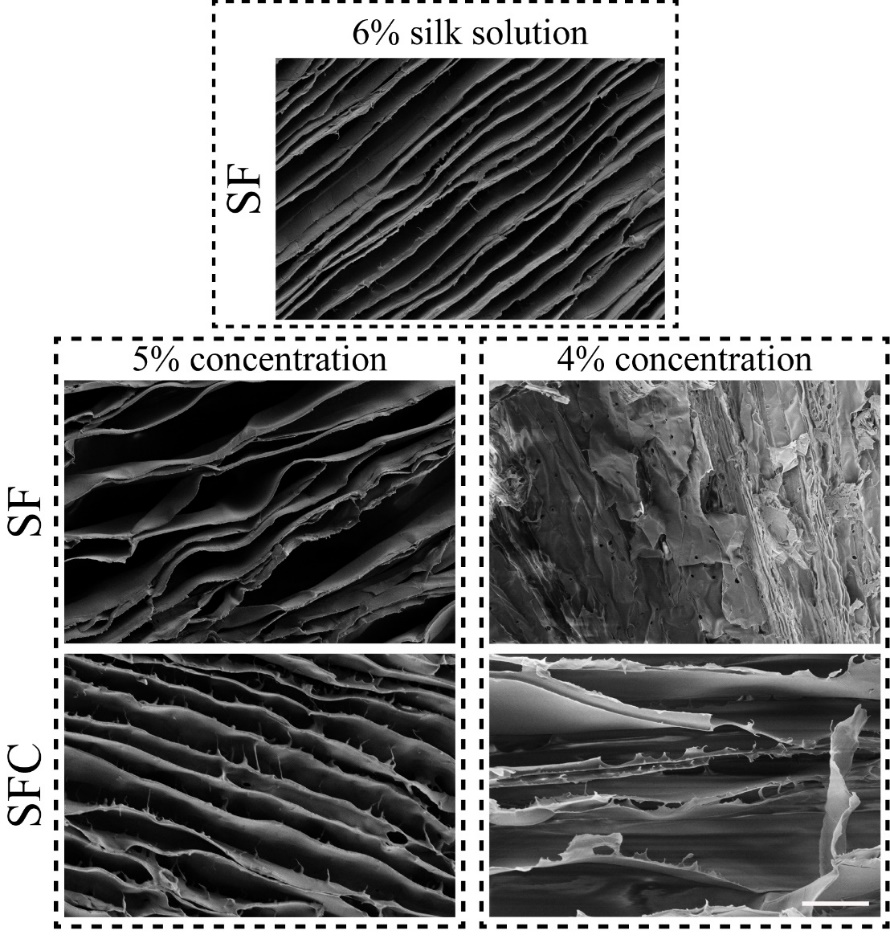


**Supplementary Figure 2**. SEM images of SF and SFC scaffolds at different concentrations (w/v). A local pore collapse was observed when the concentration of SF was reduced by 4%. Scale bars = 20 μm.


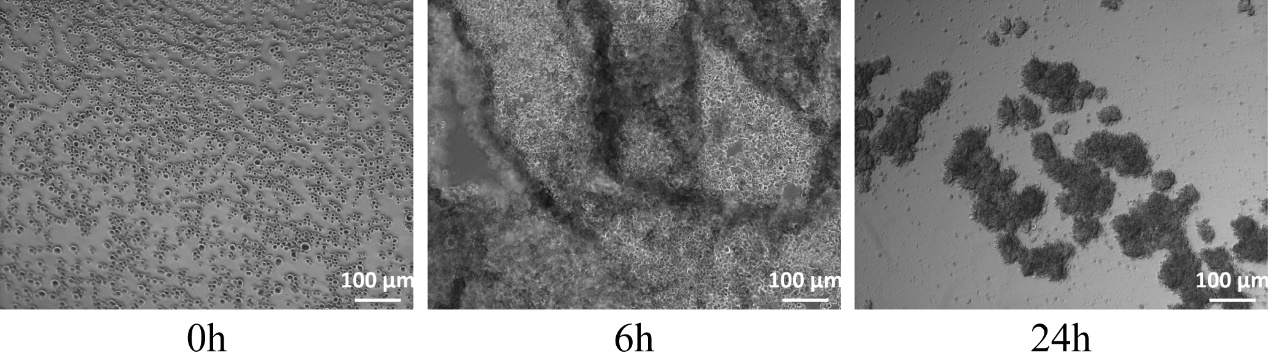


**Supplementary Figure 3**. Formation of cellular spheroids in an ultra-low attachment culture plate. Cellular spheroids with a diameter of 50–200 µm were formed within 24 h.

**Supplementary Table 1**. Preparation conditions and notations for the various scaffolds studied.

| Notation (w/v) | Preparation |
| --- | --- |
| 6% SF | 6% silk fibroin solution |
| 5% SF | Dilute 6% SF with ultrapure water to 5% |
| 4% SF | Dilute 6% SF with ultrapure water to 4% |
| 5% SFC | Dilute 6% SF with 2mg/ml collagen I to 5% |
| 4% SFC | Dilute 6% SF with 2mg/ml collagen I to 4% |

**Supplementary Table 2**. List of primers used in this study.

| **Gene** | **Forward sequence 5’-3’** | **Reverse sequence 5’-3’** |
| --- | --- | --- |
| ALB | GAGACCAGAGGTTGATGTGATG | AGTTCCGGGGCATAAAAGTAAG |
| AAT | ATGCTGCCCAGAAGACAGATA | CTGAAGGCGAACTCAGCCA |
| HNF4A | GATGTAGTCCTCCAAGCTCAC | GCCATCATCTTCTTTGACCCA |
| CYP3A4 | GTGGGGCTTTTATGATGGTCA | ACATCTCCATACTGGGCAATGA |
| CYP1A2 | CTGGGCACTTCGACCCTTAC | TCTCATCGCTACTCTCAGGGA |
| CYP2C9 | GCCTGAAACCCATAGTGGTG | GGGGCTGCTCAAAATCTTGATG |
| CYP2D6 | CCAACGGTCTCTTGGACAAAG | GGGTCGTCGTACTCGAAGC |
| MRP_2_ | GATTGCAGAGTCGCTTGAGG | GGTTGTTGCATTCGGTTCCT |
| UGT1 | TTGTCTGGCTGTTCCCACTTA | GGTCCGTCAGCATGACATCA |
| CPS-1 | AATGAGGTGGGCTTAAAGCAAG | AGTTCCACTCCACAGTTCAGA |
| G6PC | GTGTCCGTGATCGCAGACC | GACGAGGTTGAGCCAGTCTC |
